# EtMIC3 and its receptors BAG1 and ENDOUL are essential for site-specific invasion of *Eimeria tenella* in chickens

**DOI:** 10.1186/s13567-020-00809-6

**Published:** 2020-07-16

**Authors:** Wenyu Li, Mingyue Wang, Yufeng Chen, Chen Chen, Xiaoqian Liu, Xiaoting Sun, Chuanxu Jing, Lixin Xu, Ruofeng Yan, Xiangrui Li, Xiaokai Song

**Affiliations:** MOE Joint International Research Laboratory of Animal Health and Food Safety, College of Veterinary Medicine, Nanjing Agricultural University, Nanjing, 210095 People’s Republic of China

**Keywords:** *E. tenella*, EtMIC3, Site specificity, Receptor

## Abstract

Avian coccidian parasites exhibit a high degree of site specificity in different *Eimeria* species. Although the underlying mechanism is unclear, an increasing body of evidence suggests that site specificity is due to the interaction between microneme proteins (MICs) and their receptors on the surface of target host cells. In this study, the binding ability of *E. tenella* MICs (EtMICs) to different intestinal tissue was observed by immunofluorescence to identify the key surface molecule on the parasite responsible for the site specificity. Subsequently, we identified the corresponding host-cell receptors by yeast two-hybrid screening and glutathione-S-transferase pull-down experiments and the distribution of these receptors was observed by immunofluorescence in chicken intestinal tissues. Finally, we evaluated the efficacy of receptor antiserum against the infection of *E. tenella* in chickens. The results showed that EtMIC3 could only bind to the caecum while EtMIC1, EtMIC2, and EtAMA1 did not bind to any other intestinal tissues. Anti-serum to EtMIC3 was able to block the invasion of sporozoites with a blocking rate of 66.3%. The receptors for EtMIC3 were BCL2-associated athanogene 1 (BAG1) and Endonuclease polyU-specific-like (ENDOUL), which were mainly distributed in the caecum. BAG1 and ENDOUL receptor antiserum reduced weight loss and oocyst output following *E. tenella* infection, showing partial inhibition of *E. tenella* infection. These data elucidate the mechanism of site specificity for *Eimeria* infection and reveal a potential therapeutic avenue.

## Introduction

Chicken *Eimeria* are obligate intracellular parasitic protozoa that develop within intestinal epithelial cells of chickens. Infection of one or multiple *Eimeria* species causes coccidiosis which brings great economic losses to the poultry industry worldwide [1]. Currently, control of avian *Eimeria* mainly depends on usage of coccidiostats and live coccidia vaccines. However, usage of anticoccidial drugs are increasingly restricted because of drug resistance, drug residues, and legislative restrictions on in-feed drugs [2–4]. In addition, traditional live coccidial vaccines cannot be extensively applied in the poultry industry due to the increased production costs and limited production capacity of live vaccines [4, 5]. Recombinant vaccines have been shown to be a promising anticoccidial control method. The development of recombinant vaccines would be improved by better understanding of the molecular mechanism of the *Eimeria*-host interface [6–8].

Avian *Eimeria* exhibits a high degree of site specificity in the chicken intestine. *E. acervulina* develops in the duodenum, *E. maxima* parasitizes the jejunum and upper ileum, whereas *E. tenella* infects the caecum and *E. mitis* parasitizes the ileum, caeca, and rectum [5–9]. Site specificity is so strict that *Eimeria* parasite infection by intravenous, intramuscular, or intraperitoneal injections routes cause infections in the same region of the intestine as the oral route [7, 10]. Although the underlying mechanism remains unknown, site specificity seems to be determined by certain characteristics of surface molecules of the parasite and of the site itself such as molecules on the surface of intestinal cells that act as receptor or recognition sites [6, 7]. In the early stage of infection by *Eimeria*, microneme proteins (MICs) are secreted to participate in adhesion to the host cell. The process of adhesion is mediated by receptor-ligand interaction between MICs and receptor molecules on the surface of host intestinal cells [7, 11, 12]. Lai et al. [8] incubated MDBK cells with *E. tenella* sporozoite lysates and identified the cell-binding protein by western blot with rabbit serum against *E. tenella* MIC1 (EtMIC1), EtMIC2, EtMIC3, and EtMIC4. The authors found only EtMIC3 was detected in the cell-bound protein fraction. Subsequently they documented that EtMIC3 bound to α-2,3-sialyl glycan sequences present on the surface of MDBK cell through inhibition experiments in vitro and considered EtMIC3 as a major determinant of site specificity of *E. tenella* [8]. However, whether EtMIC3 is the key molecule for site specificity in the natural chicken host is unclear and its receptor in the caecal epithelium remains to be elucidated.

In the present study, we documented that EtMIC3 was a key molecule for site specificity of *E. tenella* in the natural chicken host by histological binding assay and sporozoites invasion blocking assay. Two EtMIC3 receptors distributed in chicken caeca were identified by yeast two-hybrid screening and glutathione-S-transferase (GST) pull-down experiments. The antiserum of those EtMIC3 receptors was able to inhibit the infection of chickens by *E. tenella* to some extent, revealing potential therapeutic applications.

## Materials and methods

### Animals and parasites

Newly hatched Hy-Line layer chickens were reared in sterile and coccidia-free conditions and provided with adequate feed and water without coccidiostats. Three-week-old SD rats (Qinglong Mountain Animal Breeding Farm, China) were purchased and kept in specific-pathogen-free conditions. *E. tenella* oocysts were harvested and cleaned as previously described [13]. Fresh sporulated oocysts were used in the challenge experiments. Collection of sporozoite was carried out as previously described [13].

### Expression, purification and antiserum preparations of rEtMICs

According to the sequences of EtMIC3 (FJ374765.1), EtMIC2 (FJ807654.1), EtMIC1 (EU093966.1) and EtAMA1 (JN032081.1) in GenBank, four pairs of specific primers were designed and synthesised. A Additional file 1: Table S1 shows this in more detail (see Additional file 1: Table S1). Total RNA was extracted from sporulated oocysts *E. tenella* with Total RNA Kit (Omega Bio-tek, USA) and was reverse transcribed into cDNA using First-Strand cDNA Synthesis Kit (Vazyme, China). Polymerase chain reaction (PCR) amplifications were carried out to amplify the complete open reading frames (ORFs) of EtMIC3, EtMIC2, EtMIC1, and EtAMA1 genes. The reaction mix included the cDNA of *E. tenella* (300 ng, 3 µL), forward primers (20 pmol, 1 µL), reverse primers (20 pmol, 1 µL), 2 × Taq Master Mix (25 µL, Vazyme, China), and sterile H_2_O (20 µL). The PCR amplification program was designed as follows: 94 °C for 5 min, 30 cycles (94 °C for 30 s, 60 °C for 30 s, and 72 °C for 50 s), and 72 °C for 10 min. PCR products of the four genes were cloned into the vector of pET-32a to generate prokaryotic expression plasmids pET-32a-EtMIC3, pET-32a-EtMIC2, pET-32a-EtMIC1, and pET-32a-EtAMA1 respectively. The constructed plasmids were identified by restriction enzyme digestion and sequencing. To express the recombinant proteins of EtMIC3 (rEtMIC3), EtMIC2 (rEtMIC2), EtAMA1 (rEtAMA1), and EtMIC1 (rEtMIC1), *E. coli* BL21 (DE3) transformed with the corresponding recombinant expression plasmids were cultured at 37 °C until the OD600 values reached 0.6. Then we immediately added IPTG into the culture medium with a final concentration of 1 mmol/L and continued to culture the bacteria for 5 h. Then the recombinant proteins were harvested from the bacteria and purified with His Trap^TM^ FF kit (GE Healthcare, USA) following the manufacturer’s instructions. Antiserum of rEtMIC3, rEtMIC2, rEtAMA1, and rEtMIC1 were prepared using previously described strategies [14]. Two SD rats were immunized for each recombinant protein. The titres of the antiserum were determined by ELISA assay and the serum with higher titter was used in the subsequent experiments. Simultaneously, pET-32a tag protein and its antiserum were prepared in parallel as empty vector control. Serum from normal rat was collect as negative serum control.

### Validation of the rEtMICs antisera by recognizing native EtMICs proteins from *E. tenella* sporozoites

Sporozoites (1.0 × 10^8^) were washed and suspended in 3 mL Phosphate Buffered Saline (PBS, pH 7.6). Then 30 μL of Halt™ Protease and Phosphatase Inhibitor Single-Use Cocktail (Thermo Fisher Scientific, USA) was added in sporozoites suspension and disrupted by sonication in ice (200 W, work time 5 s, interval time 10 s, 50 cycles). Subsequently the sporozoites lysate was centrifuged at 14,000 × *g* for 10 min (Eppendorf-5424R, Germany) at 4 °C, the supernatant was harvested and concentrated with a 3-kDa filter (Merck Millipore, Germany). The concentration of the protein was determined using a BCA protein assay kit (Thermo Fisher Scientific, USA). The soluble proteins were used for western blot assay to validate the quality of rEtMICs antisera.

Sporozoites protein of *E. tenella* (50 μg) were visualized on 12% SDS-PAGE and transferred to polyvinylidene difluoride membranes (Merk Millipore, Germany) for western blot analysis. The membranes were blocked in 5% skim milk in TBST (Tris-buffer saline with 0.5% Tween-20) for 2 h at 37 °C. After being washed with TBST, membranes were respectively probed with antisera against rEtMIC3, rEtMIC2, rEtAMA1 and rEtMIC1 (1:500 dilution) at 4 °C overnight. Simultaneously, antiserum against pET-32a tag protein and serum from normal rat were used as empty-vector control and negative control respectively. After three washing in TBST, membranes were incubated with goat anti-rat IgG antibody horseradish peroxidase (HRP) conjugate (1:8000 dilution, Bioworld, USA) for 1 h at 37 °C. Finally, the immunoreaction were visualized by incubating the membranes in 3,3′-diaminobenzidine tetrahydrochloride (DAB) kit following the manufacturer’s instructions (Tiangen, China).

### Binding of EtMICs to different part of chicken intestines

Intestinal tissue samples of upper, mid, and lower intestine and caecum from 2-week old chickens without coccidian infection were dehydrated, waxed, and fixed in 4% paraformaldehyde. Subsequently, they were embedded in paraffin. Tissue sections (4 µm thick) were fixed on a polylysine slides and treated with xylene twice for 10 min, absolute ethanol twice for 5 min, 95%, 85%, and 75% ethanol for 5 min consecutively, 3% hydrogen peroxide in absolute methanol for 10 min, and washed two times with PBS for 5 min. Then tissue sections were placed in sodium citrate buffer and heated to boiling (about 10 min). After cooling to room temperature, sections were covered with tris-buffered saline and polysorbate 20 (TBST) with 5% bovine serum albumin (BSA) for 2 h at 37 °C. Then tissue sections of upper, mid, and lower intestine and caecum were separately incubated with rEtMIC3, rEtMIC2, rEtAMA1 and rEtMIC1 overnight at 4 °C. Subsequently, the tissue sections were incubated with polyclonal antibodies against EtMIC3, EtMIC2, EtAMA1, and EtMIC1 (1:100) for 1 h at 37 °C, the secondary anti-rat antibodies conjugated to Cy3 at 1:1000 (Beyotime, China) for 30 min, and DAPI (Beyotime, China) for 5 min. Meanwhile, vector protein control was treated with pET-32a vector protein and incubated with its antiserum. Negative control was treated with sterile PBS and incubated with serum from an unimmunized rat. Finally, the tissue sections were detected by laser confocal microscope (Olympus, Japan).

### Inhibition of sporozoite invasion into caecum tissue by EtMIC3 antiserum

Fresh and viable *E. tenella* sporozoites were divided into three groups and treated with anti-rEtMIC3 serum, anti-pET32a vector protein serum and PBS, respectively at 4 °C for 30 min and subsequently washed three times with PBS. Meanwhile, caeca were removed immediately post-mortem from 14-day-old chickens and divided into three groups (n = 5 per group). Then the caeca were washed in 41 °C preheated HBSS buffer (PAA Laboratories, Austria) to remove the intestinal contents. Subsequently, the caeca were ligated at one end and filled with the sporozoites (10^6^ per caecum) pre-treated with the corresponding antiserum. The caeca were then ligated at the other end and incubated in PBS at 41 °C for 40 min. After that, the caeca were untied and thoroughly washed to flush out the blocked (i.e. free or loosely-attached) sporozoites and the number of the blocked sporozoites was counted. Invasion inhibition rate of each group was calculated as following: the number of blocked sporozoites/the total number of infected sporozoites.

### Establishment of cDNA library of chicken caecum

Caecal epithelial cells from 2-week chickens were excised, isolated, and purified using the method in our previous reports [15, 16]. In brief, caeca were longitudinally cut and washed with HBSS to remove the contents and mucus. The caeca were subsequently cut into small strips and incubated with 1 mmol/L DTT (Sigma, Germany) for 30 min at ambient temperature. Sequentially, the mucosal strips were incubated in 1 mM EDTA (Sigma, Germany) for 10 min at 37 °C and transferred to fresh HBSS, followed by 5–10 vigorous shakes of the container. Then the mucosal strips were removed by passing the solution over a 400 μm mesh sieve (Carl Roth, Germany). The caecal epithelial cells were isolated from single cells by an 80-μm mesh filter (Sefar, USA) through which cells such as erythrocytes, leukocytes, and fibroblasts passed easily. Subsequently, the caecal epithelial cells were cultured in extracellular matrix (ECM)-coated culture dish and cultured at 41 °C and 5% CO_2_ for 1.5 h to remove adhering cells such as fibroblasts. Finally, the purity of the caecal epithelial cells was assessed by cell alkaline phosphatase (cAKP) stain (azo coupling method) and examined by microscopy.

Total RNA of caecal epithelium cells were extracted with an RNAiso Plus Kit (TaKaRa, Japan) and treated by DNase I (TaKaRa, Japan). Construction of cDNA library was performed using the SMART cDNA Library Construction Kit (Clontech, USA). Briefly, first-strand cDNA was synthesized using oligonucleotides (Clontech, USA). Subsequently the double-stranded cDNA was purified with MiniBEST DNA Fragment Purification Kit (TaKaRa, Japan) and normalised using the TRIMMER DIRECT cDNA Normalization Kit (Evrogen, Russia). After digestion with *Sfi* I restriction enzyme, the normalised cDNA was purified with CHROMA SPIN-1000 to remove the short fragment and then ligated into pPR3-N, which was also digested with *Sfi* I restriction enzyme. Subsequently the ligation product was transformed into *E. coli* DH10B by electroporation. Finally, the transformed bacteria were plated on Luria-Bertani (LB) plates with ampicillin and thirty-two colonies were selected randomly to identify the inserts of cDNA to estimate the recombination efficiency.

### Identification of EtMIC3 receptors in chicken caecum by yeast two-hybrid system

#### Construction and expression of bait plasmid pDHB1-EtMIC3

Total RNA of sporulated oocysts of *E. tenella* was obtained using E.Z.N.A. Total RNA kit (Omega Bio-tek, USA). The cDNA was gained by reverse transcription PCR with random primers using the First-Strand cDNA Kit (TaKaRa, Japan). Amplification of target gene EtMIC3 was carried out by PCR using the cDNA as template and the specific primers (F: 5′ ATGAAGGTATACATTTGTGTCGG 3′; R: 5′ CTACAATGTGGCCCTCTCC 3′). EtMIC3 was cloned into the pDHB1 vector, forming pDHB1-EtMIC3, which was digested by *Sfi* I restriction enzyme. The bait plasmid pDHB1-EtMIC3 was transformed into NMY51 yeast cells using the Yeastmaker Yeast Transformation System 2 (Clontech, USA). Positive bait plasmid was cultured and the total protein extracted using Yeast Protein Extraction Reagent (TaKaRa, Japan). Expression of bait plasmid was detected by western blot with rEtMIC3 rat serum and rabbit anti-rat IgG as the primary and secondary antibodies, respectively. The bound antibodies were detected with the DAB Detection Kit (Beyotime, China).

#### Verification of the DUAL hunter functional assay

The bait plasmid pDHB1-EtMIC3 and the library empty plasmid pPR3-N were co-transformed into the positive pOst-Nub I and NMY51 yeast, respectively, and cultured in SD-leu-trp and SD-leu-trp-his-ade selective culture medium using the Yeastmaker Yeast Transformation System 2 (Clontech, USA). The number of colonies in different plates was counted to calculate its growth rate.

#### Library transformation and selection of interactors

The bait plasmid pDHB1-EtMIC3 and vector pPR3-N were co-transformed using the Yeastmaker Yeast Transformation System 2 (Takara, Japan) into NMY51 in SD/-Ade/-His/-Leu/-Trp including the 3-AT at concentrations of 0 mM, 10 mM, 20 mM, 40 mM, 60 mM, 80 mM, and 100 mM. Colonies were observed after 3–4 d at 30 °C. The bait plasmid pDHB1-EtMIC3 and cDNA library were co-transformed into NMY51 yeast cells. The transformed yeasts were plated on SD/-Leu/-Trp and SD/-Ade/-His/-Leu/-Trp culture plates. The colonies in the plate of SD/-Ade/-His/-Leu/-Trp were grown in the medium of SD/-Leu/-Trp and the plasmids were extracted with the Yeast Plasmid Mini Kit (Omega Bio-tek, USA) and transformed into competent cells of *E. coli* DH5α to obtain a number of plasmids. The prey plasmid and the bait plasmid pDHB1-EtMIC3 were co-transformed into yeast cells and incubated on SD/-Leu/-Trp and SD/-Ade/-His/-Leu/-Trp plates. Colonies were observed after 3–4 d at 30 °C. The presence of colonies grown in the SD/-Ade/-His/-Leu/-Trp plates indicated the existence of interaction between the bait plasmid and the prey plasmids. The prey plasmids were analysed by a basic alignment search tool (BLAST).

#### Cloning and expression of the potential EtMIC3 receptors

Total RNA was extracted from caecum from 2-week-old chickens and reverse transcribed into cDNA. Amplification of the potential receptors genes was carried out by PCR with the specific primers. A Additional file 2: Table S2 shows this in more detail (see Additional file 2: Table S2 ).The gene PCR products were ligated with pET-32a vector and the constructed plasmids were identified by enzyme digestion and sequence analysis. The correct recombinant plasmids, pET-32a-BAG1, pET-32a-SMAD5, pET-32a-CTC-487M23.8, pET-32a-ENDOUL, pET-32a-RP11-478C19.2, pET-32a-LGALS3, pET-32a-ZYX, and pET-32a-UTRN, were transformed into *BL21* (DE3) and the recombinant proteins of potential receptors were expressed and purified.

#### Verification of EtMIC3-receptor interactions by GST pull-down assay

Interaction between EtMIC3 and its potential receptors were identified by GST pull-down experiments. The gene of EtMIC3 was cloned into pGEX-6P-1 vector with GST-tag producing pGEX-6P-1-EtMIC3 recombinant protein, which was expressed and purified. The purified pGEX-6P-1-EtMIC3 protein and the eight potential EtMIC3 receptor proteins (BAG1, SMAD5, CTC-487M23.8, ENDOUL, RP11-478C19.2, LGALS3, ZYX, and UTRN) were co-incubated with GST beads separately at 4 °C for 60 min. Meanwhile, the same procedure was performed with pGEX-6P-1 vector protein as empty vector control. Then the supernatants were discarded using a magnetic frame and washed with PBS. This process was repeated three times to ensure there was non-specific absorption. The proteins of interest were eluted completely in the elution buffer. Subsequently, the collected protein was analysed by SDS-PAGE and western blot using anti-his tag monoclonal antibody. Briefly, after SDS-PAGE analysis, the proteins were transferred onto polyvinylidene fluoride (PVDF) membranes, followed by overnight blocking at 4 °C with 5% BSA. After washing, the membranes were successively incubated with anti-his tag monoclonal antibody (1:3000, Sigma Aldrich, USA) and horseradish peroxidase (HRP)-conjugated Goat anti-rat IgG (1:3000, Sigma Aldrich, USA) overnight at 4 °C. The bound antibodies were detected with ECL Chemiluminescence Detection Kit (Vazyme, China). The hydrophobic profile of the positive candidates were analysed using Protean of Lasergene 8.1.3 software (DNAStar, USA).

#### Distribution of EtMIC3 receptors in chicken intestine

Distribution of EtMIC3 receptors in chicken intestine was observed by immunofluorescence as described above. Briefly, tissue sections from upper, mid lower intestine and caecum were made and incubated with antiserum of EtMIC3 receptors BAG1 and ENDOUL (1:100) separately. Subsequently, anti-rat antibody conjugated to Cy3 was used as the secondary antibody and fluorescence was observed with a laser confocal microscope.

#### Inhibition of EtMIC3 receptor antiserum against *E. tenella* infection in chickens

EtMIC3 receptor antiserum was prepared by vaccinating chickens at two weeks of age with 200 µg of BAG1 and ENDOUL recombinant protein and pET-32a fusion protein injected intramuscularly every week for five weeks. Chickens injected with PBS served as PBS control. Ten chickens were used per group. 7 days after the last vaccination, serum was collected from the vaccinated chickens and stored at − 20 °C. Meanwhile, serum from unvaccinated chicken served as negative control.

#### Evaluation of the inhibition efficacy of the antisera against *E. tenella* infection

To evaluate the inhibition efficacy of the antiserum against *E. tenella* infection, we weighed two-week-old chickens and randomly divided them into seven groups (n = 30 per group). As shown in Table 1, two EtMIC3 receptor antiserum groups received anti-BAG1 serum and anti-ENDOUL serum while five control groups included anti-pET-32a fusion protein serum, anti-PBS serum, negative serum, challenged control, and unchallenged control. All groups were challenged orally with 5 × 10^4^*E. tenella* sporulated oocysts except for the unchallenged control. Subsequently, chickens were injected with the corresponding serum by intravenous administration every day for 7 days. 7 days after infection, chickens were weighed to document weight change. Caeca were collected and cut longitudinally. The caecal contents and mucosa were scraped, down to the caecal wall using a glass slide. Then, the caecal contents and mucosa were incubated with 1.5% trypsin at 41 °C for 30 min. Oocyst output was calculated with the MacMaster flotation method. Caecal lesion score was determined as previously described [17]. Inhibition efficacy of the receptor antiserum was evaluated based on the parameters of weight gain, oocyst output, and caecal lesion score. Group parameters were shown as mean and standard deviation (SD). Statistical significances between the different treatment groups were determined using one-way ANOVA Duncan test and were considered as significant at *p* < 0.05.

**Table 1:** **Inhibition efficacy of EtMIC3 receptor antisera against*E. tenella*infection in chickens.**

Groups	Average body weight gains (s)	Mean lesion scores (mean ± SD)	Oocyst output (lg) (mean ± SD)	Oocyst decrease ratio (%)
Anti-BAG1	28.65 ± 8.00^b^	2.86 ± 0.63^b^	6.64 ± 0.07^b^	3.63
Anti-ENDOUL	27.09 ± 7.48^b^	2.86 ± 0.81^b^	6.54 ± 0.06^b^	5.07
Anti-pET-32a	20.64 ± 8.07^c^	3.10 ± 0.92^b^	6.84 ± 0.05^c^	0.73
Anti-PBS	18.61 ± 5.26^c^	3.14 ± 0.85^b^	6.83 ± 0.07^c^	0.87
Chicken serum	19.10 ± 7.18^c^	3.16 ± 0.56^b^	6.88 ± 0.05^c^	0.15
Challenged control	13.14 ± 4.23^d^	3.16 ± 0.61^b^	6.89 ± 0.03^c^	0.00
Unchallenged control	42.19 ± 8.71^a^	0.00^a^	0.00^a^	100

In the same column, significant difference (*p* < 0.05) between numbers with different letters, no significant difference (*p* > 0.05) between numbers with the same letter.

## Results

### Preparations of recombinant proteins and antiserum of rEtMICs

**Figure 1:**
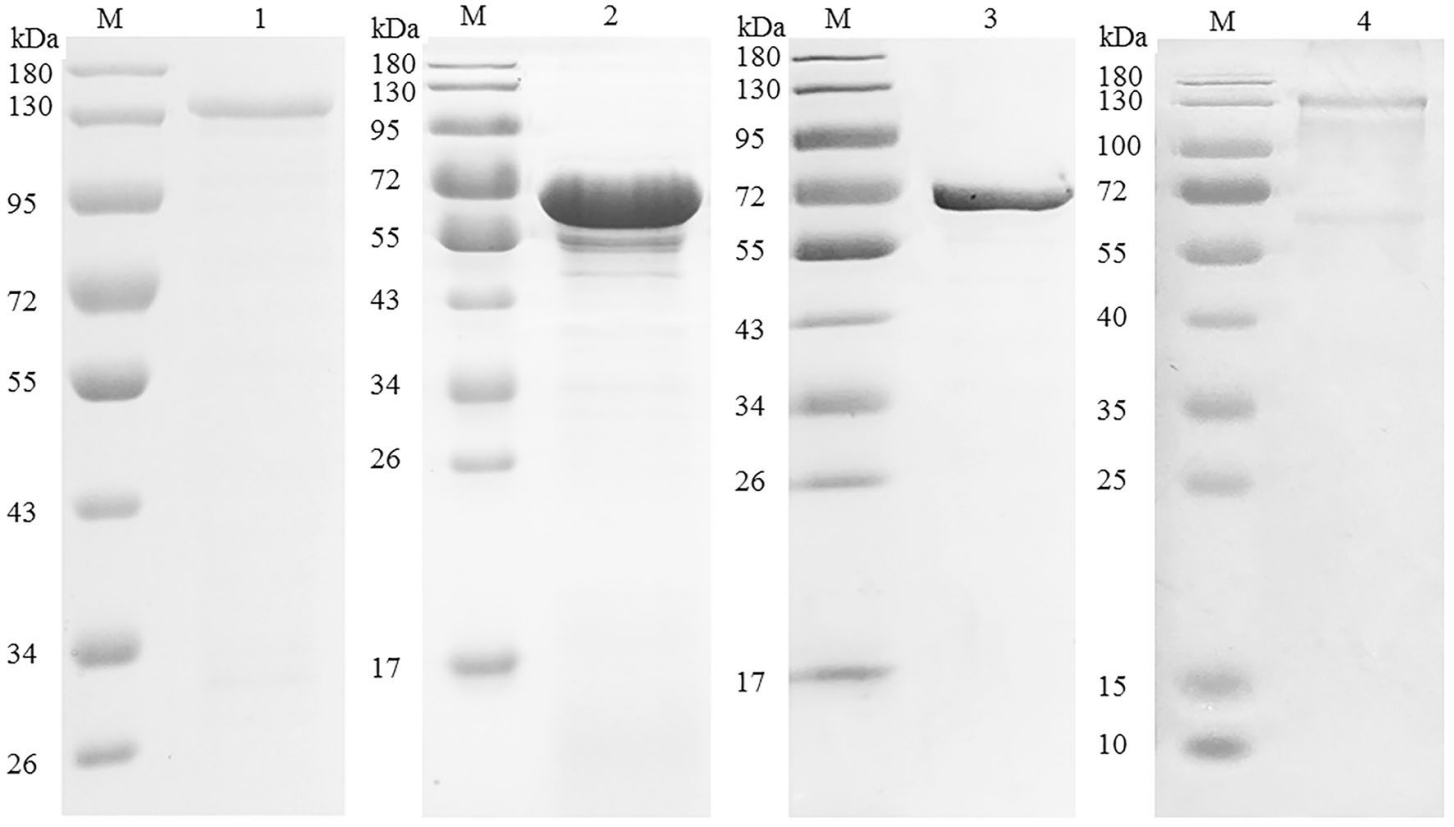
**Purification of rEtMIC3, rEtMIC2, rEtMIC1 and rEtAMA1**. M: Standard protein molecular marker. Lane 1: purified protein pET-32a-EtMIC3. Lane 2: purified protein pET-32a-EtMIC2. Lane 3: purified protein pET-32a-EtAMA1. Lane 4: purified protein pET-32a-EtMIC1.

**Figure 2:**
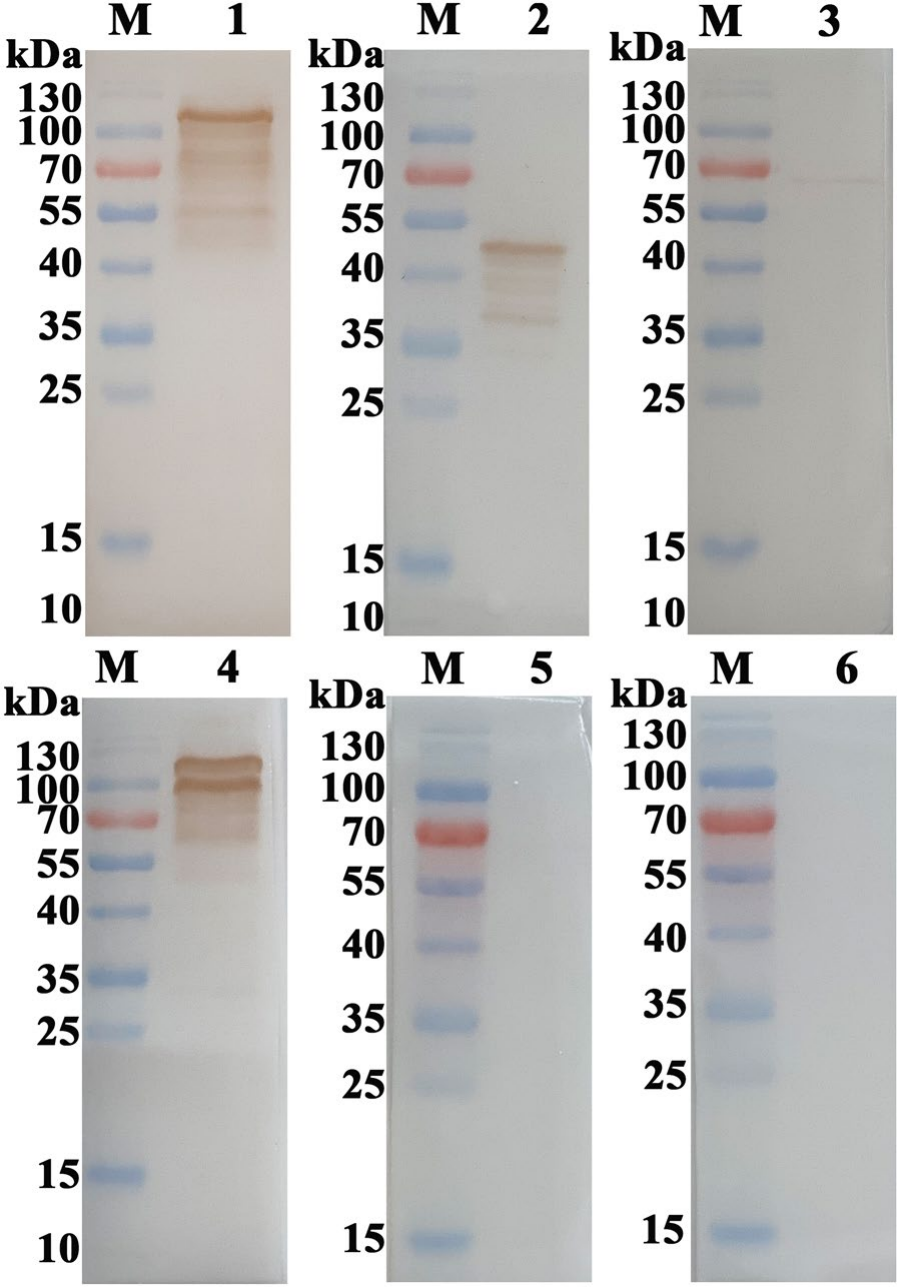
**Validation of the rEtMICs antisera by recognizing native EtMICs proteins from*E. tenella*sporozoites**. Lysate of *E. tenella* sporozoites was analyzed by western blot using antisera of rEtMIC3, rEtMIC2, rEtAMA1 and rEtMIC1 as primary antibody separately. Antisera of rEtMIC3 (lane 1), rEtMIC2 (lane 2), rEtAMA1 (lane 3) and rEtMIC1 (lane 4) recognized the corresponding native proteins from the lysate of *E. tenella* sporozoites respectively. The negative serum (lane 5) and pET-32a tag protein antiserum (lane 6) did not reacted with any native protein of *E. tenella* sporozoites. Lane M, standard protein molecular marker.

Recombinant proteins of EtMIC3, EtMIC2, rEtAMA1 and EtMIC1 were harvested from *E. coli* host. After purification, SDS-PAGE analysis revealed three prominent bands with sizes of 126, 72, and 95 kDa, equal to the molecular weights of rEtMIC3, rEtMIC2 and rEtAMA1, respectively (Figure  1). As for rEtMIC1, the purified band was about 130 kDa, which closely approximated the molecular weight of EtMIC1 dimer. The purified bands could be recognized by anti-his tag monoclonal antibody. These results indicated that the four recombinant proteins were expressed and well purified. Antiserum of rEtMIC3, rEtMIC2, rEtAMA1, rEtMIC1 and pET-32a tag protein were obtained from rats vaccinated with the corresponding recombinant proteins and ELISA assay revealed that their titres were 2^13^, 2^18^, 2^18^, 2^19^ and 2^18^ respectively. The quality of rEtMICs antisera was validated by recognizing native EtMICs proteins from *E. tenella* sporozoites using western-blot analysis. As shown in Figure  2, western-blot analysis indicated that antisera of rEtMIC3, rEtMIC2, rEtAMA1 and rEtMIC1 reacted with the corresponding native proteins from the lysate of *E. tenella* sporozoites respectively. Meanwhile, the negative serum and pET-32a tag antiserum did not reacted with any native protein of *E. tenella* sporozoites.

### Binding of rEtMIC3, rEtMIC2, rEtAMA1 and rEtMIC1 to different parts of chicken intestines

**Figure 3:**
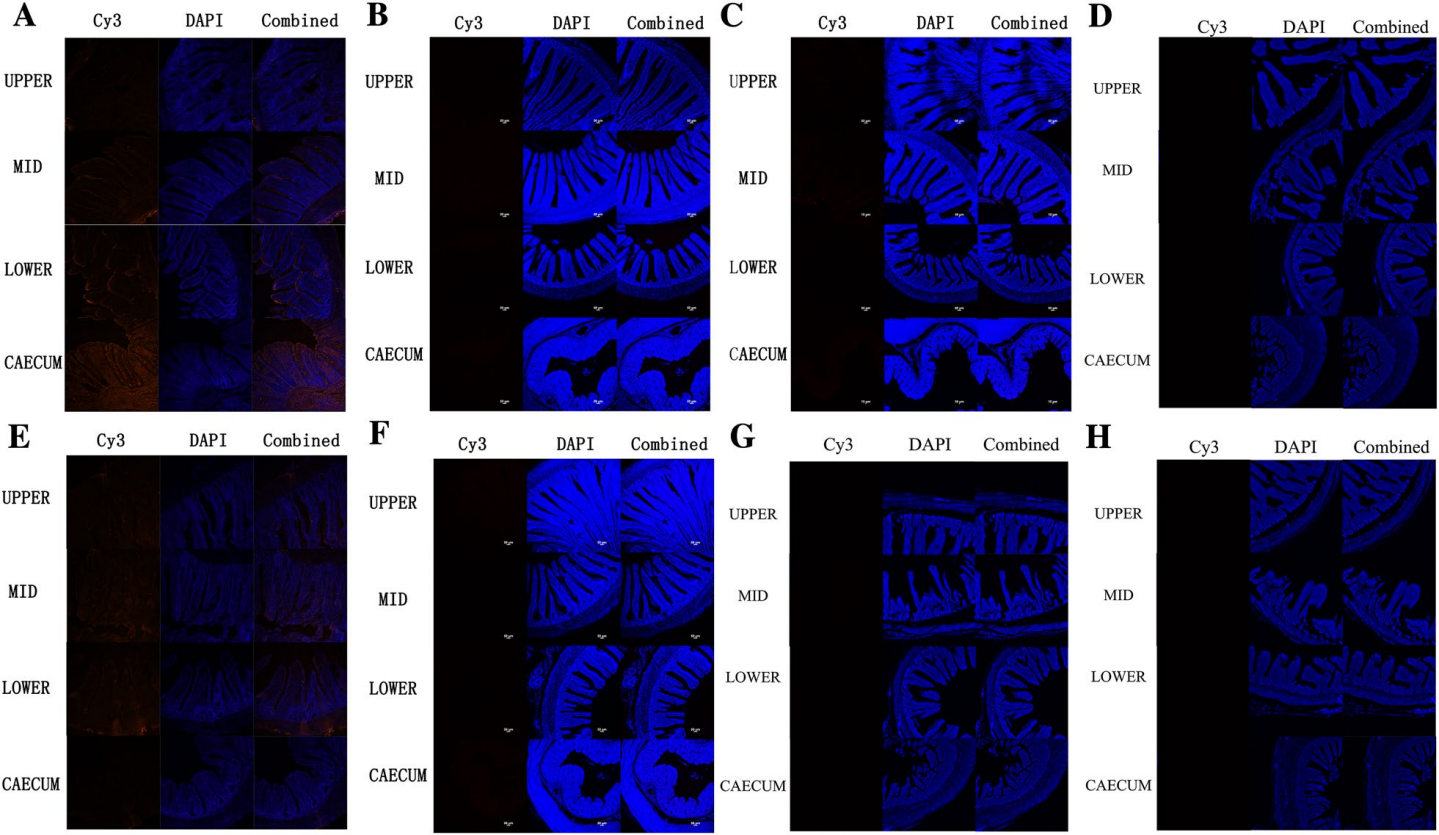
**Binding of rEtMIC3, rEtMIC2, rEtAMA1 and rEtMIC1 to upper, mid, lower intestine and caecum of chicken intestines.** Histological sections from upper, mid, lower intestine and caecum of chickens were incubated with rEtMIC3, rEtMIC2 rEtAMA1 and rEtMIC1 separately and then incubated with the corresponding antisera of the recombinant proteins separately. The vector protein control was treated with pET-32a vector protein and incubated with its antiserum. The negative control was treated with sterile PBS and incubated with serum from unimmunized rat. The binding ability was observed by immunofluorescence assay, and obvious red fluorescence was considered as positive. **A** rEtMIC3 specifically bound to caecum, but not other parts of the intestine of chickens. rEtMIC2 (**B**) rEtAMA1 (**C**) and rEtMIC1 (**D**) did not bind to any part of chicken intestine. (**E, F, G**) pET-32a vector protein (vector protein control) did not bind to any part of chicken intestine. **H** No obvious red fluorescence was observed in negative control.

To observe the binding of rEtMIC3, rEtMIC2, rEtAMA1 and rEtMIC1 to different part of chicken intestines, histological sections taken from upper, mid, and lower intestine and caecum of chickens were incubated with rEtMIC3, rEtMIC2, rEtAMA1 or rEtMIC1 and then incubated with the corresponding antiserum of the recombinant proteins. The binding ability was observed by immunofluorescence assay. The binding ability of rEtMIC3 is shown in Figure  3A, strong red fluorescence was observed only in the caecum tissue compared to the upper, mid, and lower intestinal tissues, which suggested that rEtMIC3 specifically bound to caecum, but not other parts of the intestine. In contrast, no obvious fluorescence was detected in any section of the intestinal tissue incubated with rEtMIC2, rEtAMA1or rEtMIC1 (Figure  3B–D), suggesting that rEtMIC2, rEtAMA1, and rEtMIC1 did not bind to any part of the chicken intestine. There was no obvious red fluorescence in any section of the intestinal tissue of the pET-32a vector and PBS controls (Figure  3E–H). Blocking assay indicated that the rEtMIC3 antiserum clearly inhibited the invasion of sporozoite into caecal tissue compared to the control serum. The invasion inhibition rate of rEtMIC3 antiserum was 66.3%. These results indicated a key role for EtMIC3 in site specificity of *E. tenella* infection in chickens.

### Establishment of cDNA library of chicken caecum

Total RNA was extracted from epithelial cells of chicken caecum to construct a yeast two-hybrid system. The result showed that the total RNA was intact and the titres of the primary cDNA library and the amplified one were 3 × 10^6^ CFU/ml and 3 × 10^9^ CFU/ml, respectively. The length of inserts varied between 400 and 2000 bp with an average length more than 1000 bp. A Additional file 3: Figure S1 material shows this in more detail (see Additional file 3: Figure S1). These data showed that the cDNA library of chicken caecum was successfully constructed and could be used for screening of potential receptors interacting with EtMIC3.

### Construction of bait plasmid pDHB1-EtMIC3 and identification of its expression

**Figure 4:**
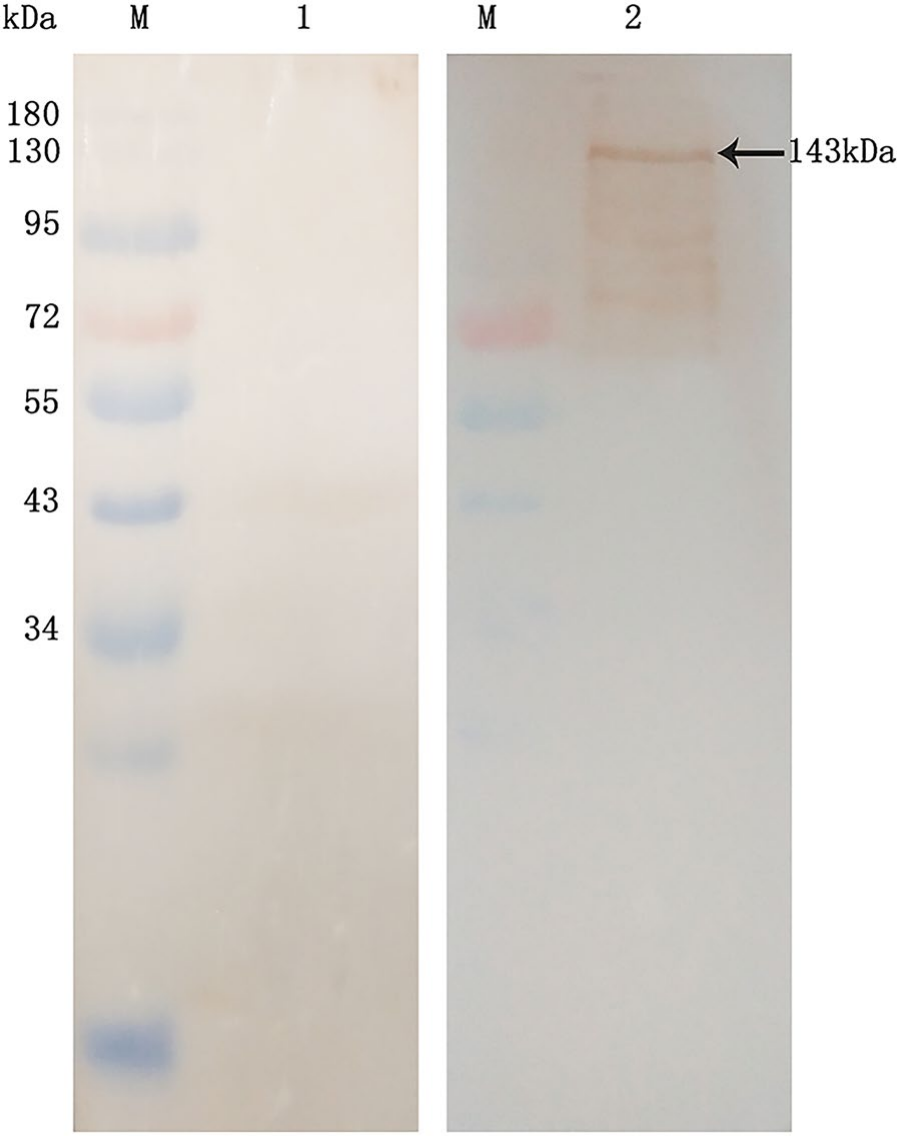
Detection of pDHB1-EtMIC3 expression in yeast by Western blot. M: standard protein marker. Lane 1: negative control. Lane 2: pDHB1-EtMIC3 expressed in NMY51 yeast.

Bait plasmid pDHB1-EtMIC3 were constructed and transformed into NMY51 yeast. Total protein was extracted and identified with anti-EtMIC3 serum by western blot. A band was apparent at 143 kDa, which was the same as protein pDHB1-EtMIC3 (Figure  4). The results indicated that bait plasmid pDHB1-EtMIC3 was expressed in NMY51 yeast.

### Verification the DUAL hunter functional assay

**Figure 5:**
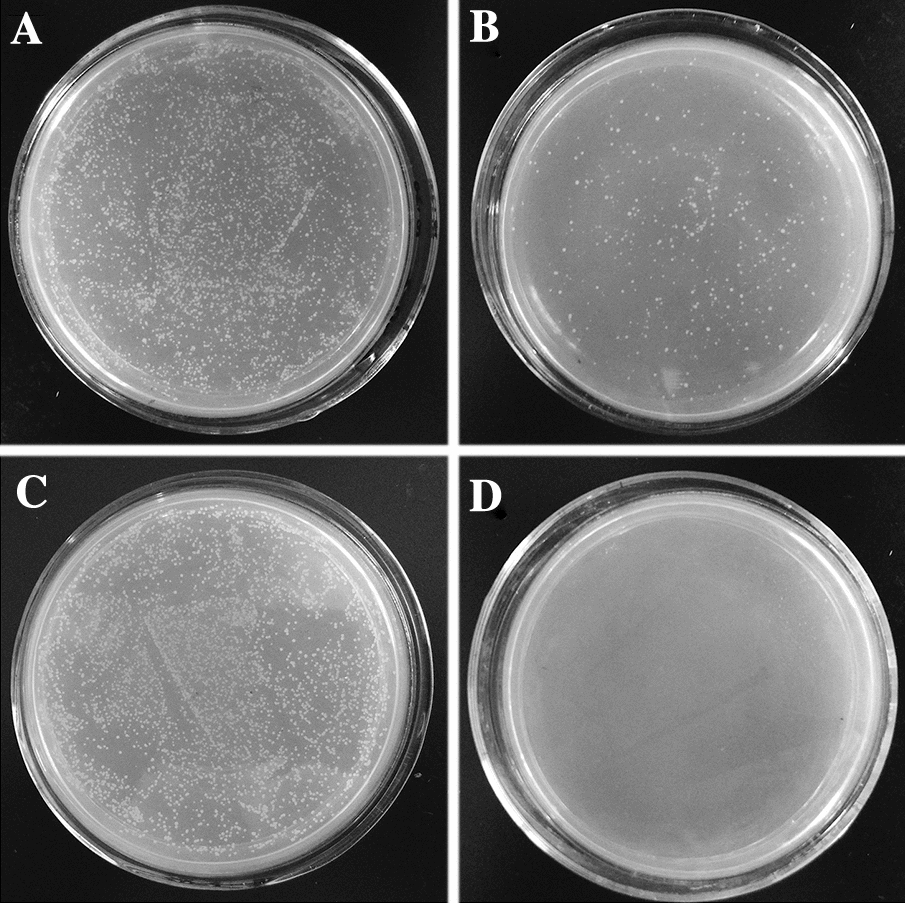
**The DUALhunter functional assay**. **A** Transformation with bait plasmid and pOst1-NubI in NMY51 grown in SD-2 plate. **B** Transformation with bait plasmid and pOst1-NubI in NMY51 grown in SD-4 plate. **C** Transformation with bait plasmid and pPR3-N grown in SD-2 plate. **D** Transformation with bait plasmid and pPR3-N grown in SD-4 plate.

pOst1-NubI and pPR3-N were transformed with pDHB1-EtMIC3 into NMY51 yeast and the number of colonies in SD/-Leu/-Trp and SD/-Ade/-His/-Leu/-Trp plates were counted to calculate the growth rate. The growth of pOst1-NubI and pDHB1-EtMIC3 averaged 10.63% with a range of 10–100% (Table 2), which indicated that there was an interaction between pOst1-NubI and pDHB1-EtMIC3, and bait plasmid pDHB1-EtMIC3 could be used for the DUAL hunter system. However, the growth of pPR3-N and pDHB1-EtMIC3 was 0%, which revealed that there was a non-specific binding between pPR3-N and pDHB1-EtMIC3 (Figure  5). Therefore, there was no need to add 3-AT to inhibit non-specific binding.

**Table 2:** **The number of colonies on plates and the percentage of growth on selective plates.**

Samples	Colonies in SD-trp-leu	Colonies in SD-trp-leu-his-ade	Growth rate( %)
pDHB1-EtMIC3 + pRR3-N	4684	0	0
pDHB1-EtMIC3 + pOst1-NubI	3564	379	10.63

### Verification of the positive prey plasmids by retransformation with pDHB-EtMIC3 into yeast cells

**Figure 6:**
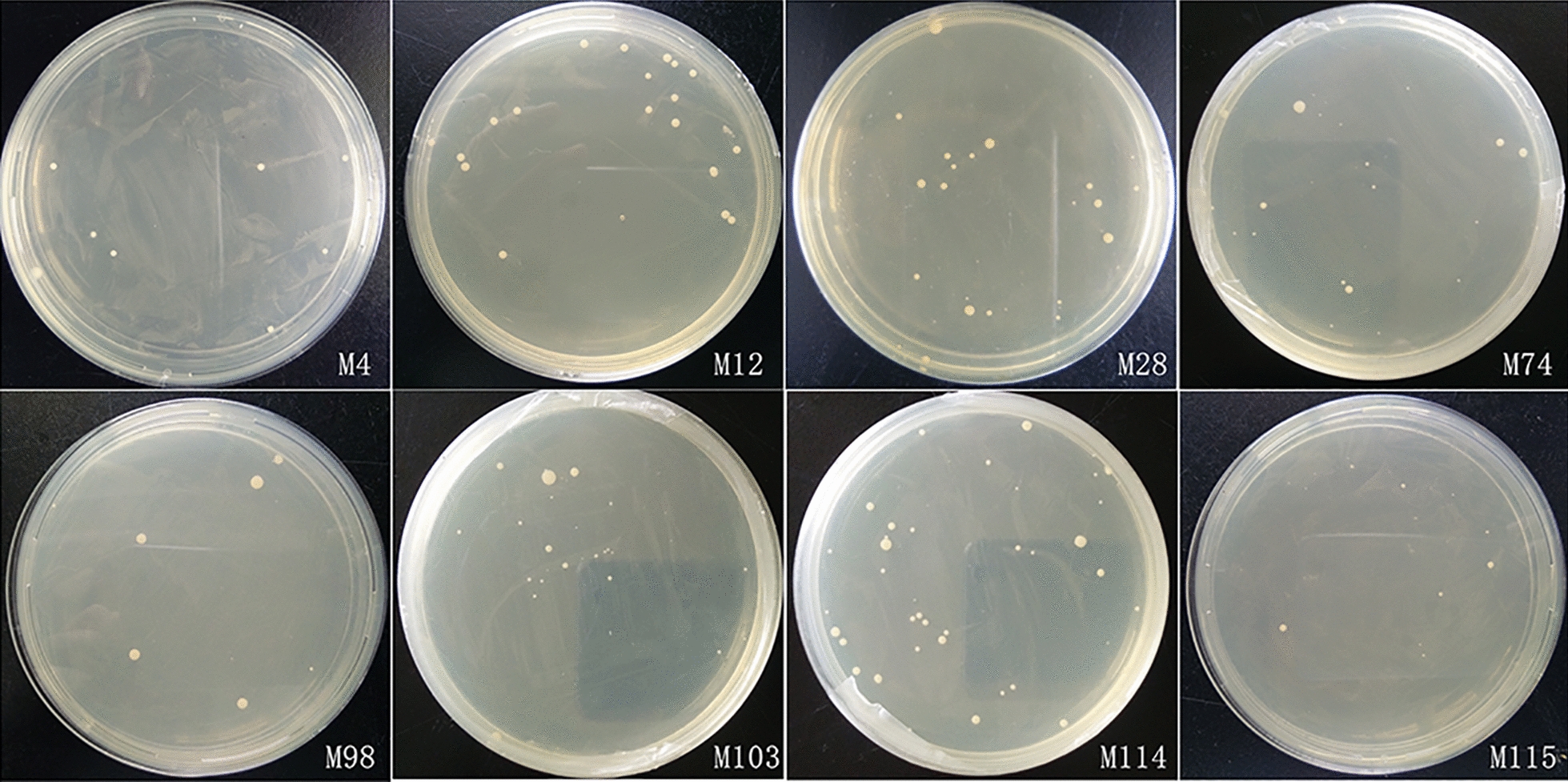
**Verification of the positive prey plasmids by retransformation with pDHB-EtMIC3 into yeast cells**.

To verify the positive prey plasmids, they were retransformed with pDHB-EtMIC3 into yeast cells. As shown in Figure  6 and Additional file 4: Table S3, eight positive prey plasmid transformed yeasts grew well on the selective medium plates, which indicated that the prey plasmids did interact with EtMIC3 and could be considered as the potential receptors of EtMIC3. The eight genes were sequenced and analysed through the NCBI website (Table 3). They encoded anaphase promoting complex subunit 7 (RP11-478C19.2), lectin, galactoside-binding, soluble 3 (LGALS3), BCL2-associated athanogene 1 (BAG1), Zyxin (ZYX), SMAD family member 5 (SMAD5), Utrophin (UTRN), endonuclease polyU-specific-like (ENDOUL), and one uncharacterised protein.

**Table 3:** **Gene bioinformatics analysis of positive prey plasmids.**

Genes	ORF (bp)	Amino acid (aa)	Molecular weight (kDa)	PI	Transmembrane region	Signal peptide	Glycosylation site	Phosphorylation site
RP11-478C19.2	1698	565	63.034	5.53	NO	NO	1	56
LGALS3	678	225	24.108	8.56	NO	NO	0	9
BAG1	630	209	23.209	6.13	NO	NO	0	9
ZYX	1629	542	58.538	6.98	NO	NO	3	59
SMAD5	1398	465	52.228	7.64	NO	NO	4	49
UTRN	2829	942	107.820	6.27	NO	NO	3	88
ENDOUL	870	289	33.582	6.43	1	NO	2	21
CTC-487M23.8	435	144	16.099	9.83	NO	NO	1	12

### Verification of the identified receptors of EtMIC3 by GST pull-down

**Figure 7:**
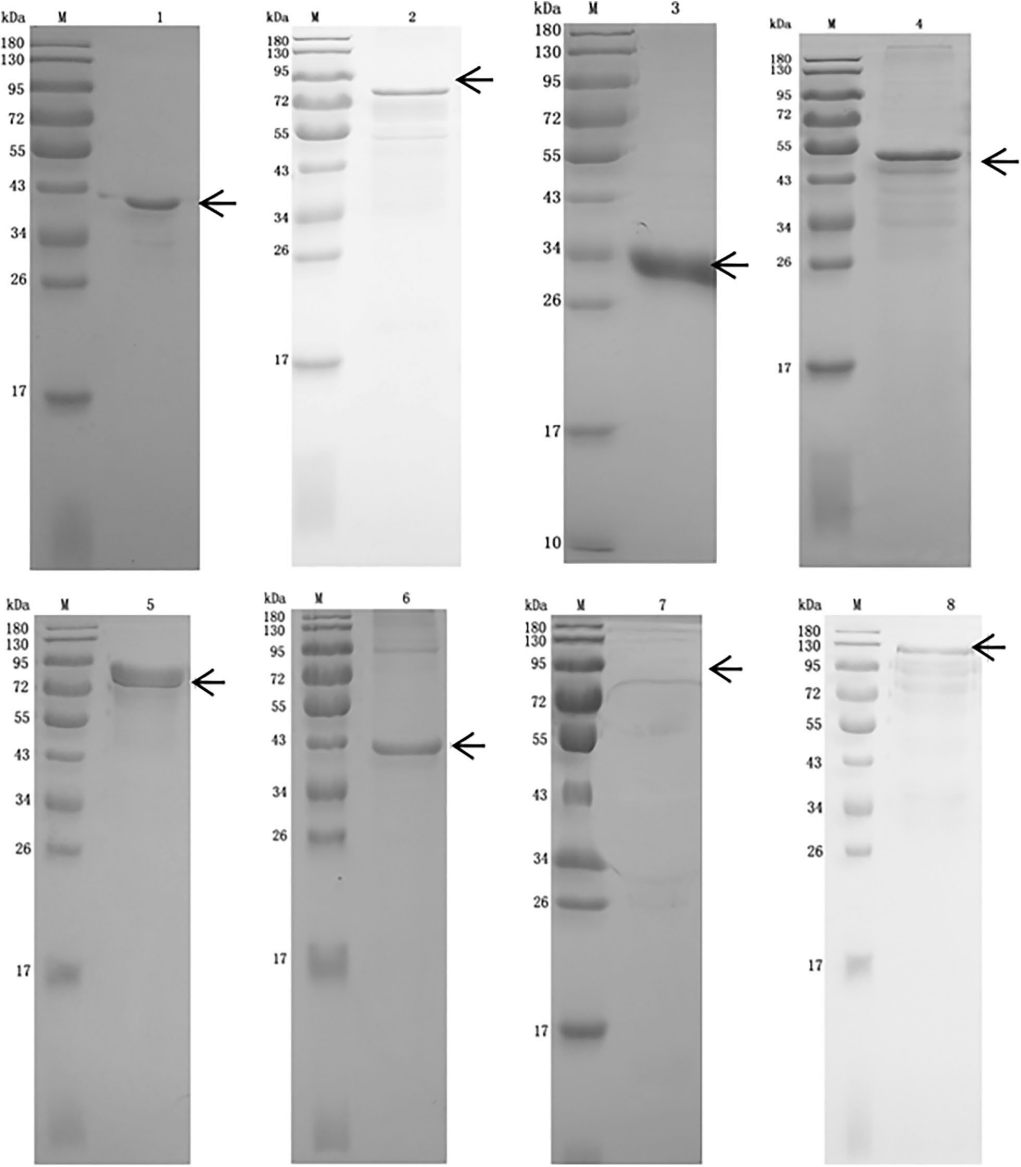
**Purification of the 8 potential EtMIC3 receptors**. M: standard protein molecular marker. Lane 1: purified protein pET-32a-BAG1. Lane 2: purified protein pET-32a-SMAD5. Lane 3: purified protein pET-32a-CTC-487M23.8. Lane 4: purified protein pET-32a-ENDOUL. Lane 5: purified protein pET-32a-RP11-478C19.2. Lane 6: purified protein pET-32a-LGALS3. Lane 7: purified protein pET-32a-ZYX. Lane 8: purified protein pET-32a-UTRN.

**Figure 8:**
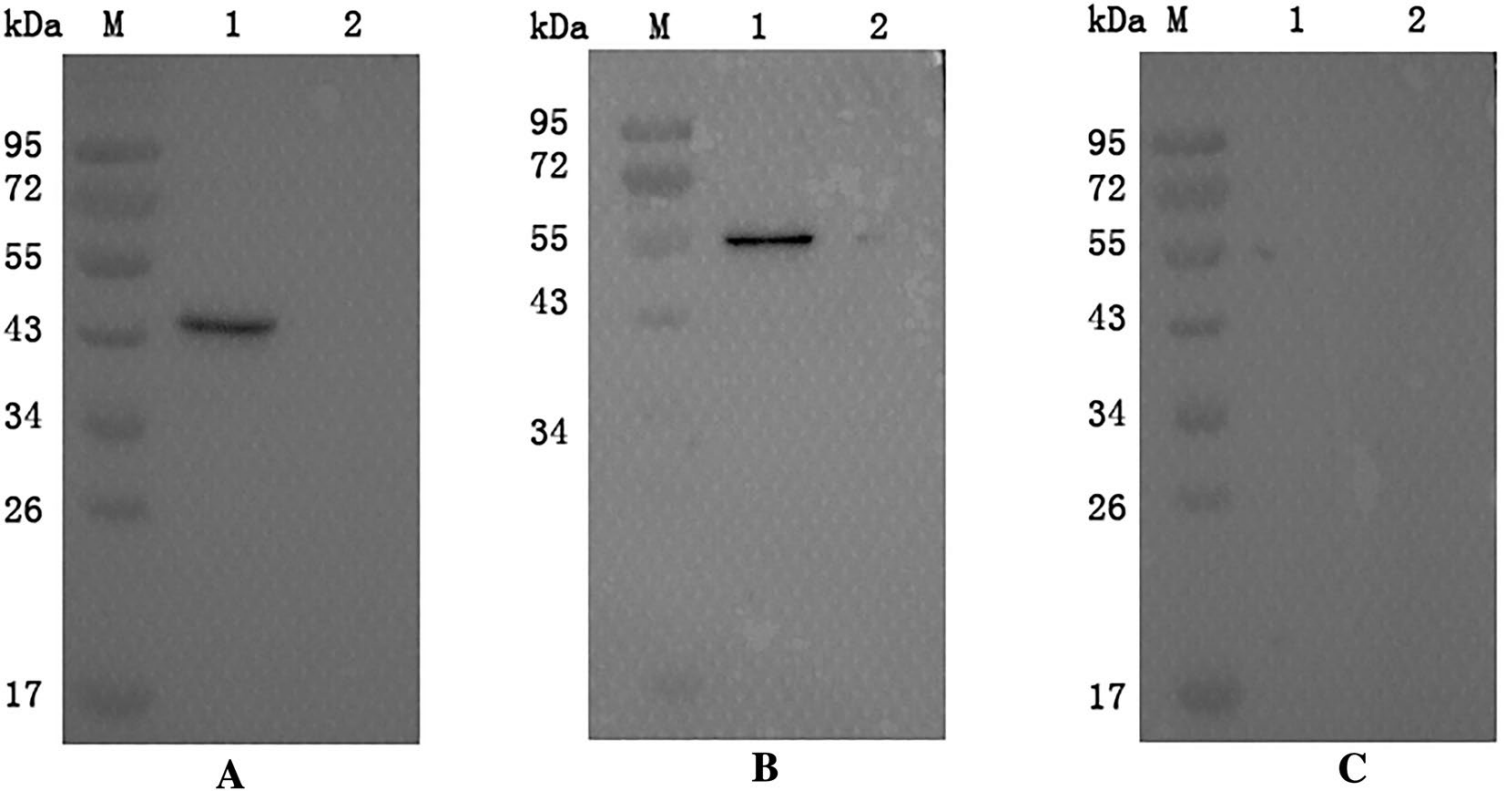
**Verification of the identified potential receptors of EtMIC3 by GST pull-down assay**. M: standard protein molecular marker. **A** Verification of the interaction between EtMIC3 and BAG1. Lane 1: pGEX-6p-1-EtMIC3 and BAG1. Lane 2: pGEX-6p-1 vector protein and BAG1. **B** Verification of the interaction between EtMIC3 and ENDOUL. Lane 1: pGEX-6p-1-EtMIC3 and ENDOUL. Lane 2: pGEX-6p-1 vector protein and ENDOUL. **C** Detection of the interaction between EtMIC3 and pET-32a vector protein. Lane 1: pGEX-6p-1-EtMIC3 and pET-32a vector protein. Lane 2: pGEX-6p-1 vector protein and pET-32a vector protein.

The eight potential receptors were expressed and purified (Figure  **7**). SDS-PAGE revealed purified recombinant proteins of BAG1, SMAD5, CTC-487M23.8, ENDOUL, RP11-478C19.2, LGALS3, ZYX, and UTRN with the corresponding sizes of 41, 70, 34, 51, 81, 42, 76, and 125 kDa. Subsequently, the interactions between EtMIC3 and its eight potential receptors were verified by GST pull-down assay. As shown in Figure  8, the interactions of EtMIC3-BAG1 and EtMIC3- ENDOUL were recognized by anti-his antibody with bands at 41 and 51 kDa, respectively, indicating that there was interaction between EtMIC3 and its receptors of BAG1 and ENDOUL. The result verified that the receptors of EtMIC3 were BAG1 and ENDOUL present on caecal epithelial cells of chickens.

A Additional file 5: Figure S2 shows the hydrophobic profile of candidate ENDOUL and BAG1. A typical transmembrane helix consists of 20–30 hydrophobic amino acids [18, 19]. As shown in Additional file 5: Figure S2A, ENDOUL possessed one hydrophobic region composed of 26 hydrophobic amino acids (residues 224 to 249), suggesting a possible transmembrane domain. TMpred (https://embnet.vital-it.ch/software/TMPRED_form.html) prediction revealed that there was a possible transmembrane helix (residues 220 to 247) in ENDOUL. Hydrophobic profile of BAG1 was shown in Additional file 5: Figure S2B. Although there were seven hydrophobic regions, no region composed of hydrophobic amino acids more than 20 residues was predicted in BAG1. TMpred prediction did not reveal any obvious transmembrane helix in BAG1. However, a potential glycosylphosphatidylinositol (GPI)-modification site (residue 192) was predicted in BAG1 by big-PI Predictor (http://mendel.imp.ac.at/sat/gpi/gpi_server.html#opennewwindow), suggesting that BAG1 probably was a GPI-anchored receptor.

### Distribution of EtMIC3 receptors BAG1 and ENDOUL in chicken intestine

Immunofluorescence assay was performed to determine the distribution of EtMIC3 receptors BAG1 and ENDOUL in chicken intestine. A Additional file 6: Figure S3 shows this in more detail (see Additional file 6: Figure S3). The distribution of BAG1 is shown in Additional file 6: Figure S3, weak red fluorescence was observed only in the caecum tissue incubated with BAG1 antiserum with an absence of fluorescence in any other part of the intestine. Meanwhile, strong red fluorescence was observed in the caecum tissue incubated with ENDOUL antiserum as well (Additional file 6: Figure S3B). There was no obvious fluorescence in any sections of the intestinal tissue incubated with antiserum of pET-32a vector protein (Additional file 6: Figure S3**C**). The result showed the receptors BAG1 and ENDOUL are distributed in the caecum and not in other places of the intestine.

### Evaluation of the inhibition efficacy of EtMIC3 receptor antiserum against *E. tenella* infection in chickens

The inhibition efficacy of EtMIC3 receptor antiserum against *E. tenella* infection was evaluated by challenge with *E. tenella* in chickens. Table 1 indicated that injections of anti-BAG1 serum and anti- ENDOUL significantly decreased the oocyst output and increased the weight gains compared with the PBS control and anti-pET-32a fusion protein serum (*p* < 0.05). While the injections did not significantly alleviate caecal lesions caused by infection of *E. tenella*, this result indicated that anti-BAG1 serum and anti-ENDOUL serum could partially inhibit infection of *E. tenella* in chickens.

## Discussion

Although invasion and replication of chicken *Eimeria* species is restricted to epithelial cells of the intestine in chickens, different species invade different regions of the intestine, exhibiting rigorous site-specificity for invasion. For example, *E. acervulina*, *E. maxima*, and *E. tenella* parasitize in the upper and middle intestine and caecum, respectively, while *E. mitis* parasitizes the lower intestine and rectum. Interestingly, *E. necatrix* invades in the middle of the intestine and subsequently transfers to the caecum to develop into oocysts [5–9]. Although the exact mechanism remains unknown, it has been suggested that the site specificity of *Eimeria* species may be determined by molecules present on both intestinal cells of the chicken and the invading stage of the parasite [6, 7]. In this study, EtMIC3 was further documented to be a key molecule for site specificity of *E. tenella*, which interacted with the two receptors BAG1 and ENDOUL on caecal cells of the natural chicken host. These results contribute to elucidate the exact mechanism for site specificity of *Eimeria* species.

*Eimeria* species share a conserved mode of invasion with other apicomplexan parasites which includes the initial recognition, attachment, and invasion of target host cells [7–12, 20]. The initial recognition and attachment of host cell is governed by MICs. In other words, the first specific high-affinity interaction between *Eimeria* parasite and host cell is conferred by MICs. Therefore the site specificity of *Eimeria* species is likely to be determined largely by their repertoires of expressed MICs [14]. In the last few years, an increasing number of MICs have been identified and characterised from various apicomplexan protozoa [21–23]. In *Eimeria* species, at least eleven MICs have been reported, namely MIC1-7 and AMA 1-4 (apical membrane antigen 1- 4) [8, 24–27]. Lai et al. [8] found that only EtMIC3 was detected in MDBK cell-bound protein fraction by western blot with rabbit serum against EtMIC1, EtMIC2, EtMIC3, and EtMIC4, based on which they observed the binding ability of EtMIC3 to different parts of chicken intestine and found that binding of EtMIC3 was restricted to caecal epithelium, but not in other parts of the chicken intestine. In this study, we compared the binding ability of EtMIC1, EtMIC2, EtMIC3, and EtAMA1 to different parts of the chicken intestine and similarly, found that only EtMIC3 could bind to caecum, while EtMIC1, EtMIC2, and EtAMA1 did not bind to any part of the chicken intestine. The results of our study combined with data from Lai et al. [8] suggest that EtMIC3 is a key molecule for site specificity of *E. tenella* in chickens.

It has been documented that molecules on host cells serve as specific receptors or recognition sites for *Eimeria* infection. Receptor-ligand interaction between MICs and their specific receptors on host cells mediates site recognition and cell adhesion during cell invasion by *Eimeria* parasites [11, 28, 29]. However, the specific receptors of MICs on host cells remain unknown. In this study, we identified BAG1 and ENDOUL as the specific receptors for EtMIC3 on host cells. Antiserum of EtMIC3 receptors was observed to block *Eimeria* invasion to some extent, which suggested that BAG1 and ENDOUL were important receptors of EtMIC3 for *Eimeria* invasion. Interesting, we observed that distribution of receptors BAG1 and ENDOUL was restricted in the caecum, which may explain caecum tropism of *E. tenella*. The relative distribution of host cell receptors has also been reported in other apicomplexan protozoa. For example, Nesterenko et al. [30]. reported that putative host cell receptors of CP47 distributed in ileal tissue with a higher concentration than in duodenal tissues, explaining the ileal tissue tropism of *Cryptosporidium parvum* in neonatal mice. Our result strongly supports the hypothesis that the relative distribution of host cell surface molecules contributes to site specificity for invasion by *Eimeria* species, although several other hypotheses explaining the site specificity for invasion by *Eimeria* parasites have been proposed [7].

BAG1was originally reported as a protein that bound to mouse Bcl2 and increased the anti-apoptosis properties. BAG1 was then identified as a member of steroid receptor superfamily and glucocorticoid receptor (GR)-binding protein [31]. Mata-Greenwood et al. [32] reported the binding of BAG1 to immature GR (glucocorticoid receptor) complex could repress GRα transactivation. Chun et al. [31] revealed a novel role for BAG1 as an additional intracellular-binding protein and nuclear chaperone for vitamin D metabolites. These findings suggest that BAG1 modulates various cellular processes by interaction with multiple cellular molecules. Hence, it is rational that BAG1 was identified as a candidate receptor of EtMIC3 in the current study. However, it is unexpected that ENDOUL, a nuclease, was identified as a candidate receptor of EtMIC3 in our study. Some previous reports might support our finding. Vanamee et al. [33] revealed two glucocorticoid receptor-like Zn(Cys)_4_ motifs in the α subunit of Bsl I restriction endonuclease using X-ray absorption spectroscopic analysis. The authors proposed that one of the Zn(II) motifs may mediate protein-DNA interactions and the other might mediate protein–protein interactions. Curtis et al. [34] reported that apurinic/apyrimidinic endonuclease 1 (Ape1) interacted with estrogen receptor (ER) and promoted the interaction of ER with estrogen-response elements (EREs) in DNA. However, better understanding on the role of the two identified receptors in site specificity will derive from further researches.

The hydrophobic profile of candidate ENDOUL revealed that it had a possible transmembrane domain, suggesting that ENDOUL probably was a transmembrane receptor of EtMIC3. However, the situation was different for BAG1. Although BAG1 lacked obvious transmembrane domain, it had a GPI-modification site, suggesting that BAG1 probably was a GPI-anchored receptor of EtMIC3. Actually, an increasing number of pathogens appear to utilize GPI-anchored receptors to infect host cells [35]. For example, during the infection of Avian Sarcoma and Leukosis Virus (ASLV), both the transmembrane receptor (TVA950) and the GPI-anchored receptor (TVA800) supported virus infection [36]. HYAL2 has been proved as a GPI-anchored cell-surface receptor for jaagsiekte sheep retrovirus [37]. Thus, ENDOUL and BAG1 probably act as the transmembrane receptor and the GPI-anchored receptor of EtMIC3 during invasion of *E. tenella*. Nevertheless, these were just the results predicted by softwares. The real situation needs to be verified by further experiments.

In this study, although BAG1 and ENDOUL were determined as host receptors of EtMIC3 for *Eimeria* invasion, their antiserum did not completely block subsequent infection by *E. tenella* parasite in vivo. One explanation could be that host cell receptors may be only one of many factors for the attachment and invasion of host cells by *Eimeria* parasites. Other factors such as cytoskeletal composition and membrane fluidity may have strong influence on the invasion process. In addition, host cell receptor molecules have many other biological functions. For example, BAG1 participates in various biological functions such as signal pathways, cellular proliferation, apoptosis, transcription, differentiation, embryogenesis, and neoplasia [38]. ENDOUL is a multi-functional protein involved in DNA repair [39].

When we evaluated the inhibition efficacy of EtMIC3 receptor antiserum against *E. tenella* infection in chickens, we found that lesion scores of the treatment groups did not show statistical differences, while oocyst output and weight gain were statistically different from the control groups, suggesting that lesion scores did not correlate well with weight gain and oocyst output. A similar finding has been reported previously. Conway et al. [40] evaluated the relationship of coccidia lesion scores and weight gain in *E. acervulina*, *E. maxima*, and *E. tenella* infections and found that high lesion scores caused by the three species were associated with only minor changes in weight gain in medicated birds compared to nonmedicated birds. Chapman et al. [41] stated that enteric lesions may be present even though weight gain is not depressed in partially or completely immune birds. Although lesion scoring has been widely used for quantitative evaluation of the experimental treatments effect on the severity of coccidia infections, it has substantial limitations for evaluating anticoccidial efficacy [40]. Lesion scoring does not fully reflect the disease severity in induced infection and does not correlate well with weight gain and oocyst output. Chapman et al. [41] proposed that lesion scoring should not be mandatory for vaccine evaluation and should not be evaluated in isolation, but should rather be correlated with other criteria including weight gain, feed conversion efficiency, and oocyst production. Hence, we measured the weight gain, oocyst output, and lesion scores to evaluate the inhibition efficacy of EtMIC3 receptor antiserum. Although lesion scores did not correlate well with either weight gain and oocyst output, the latter two correlated well with each other, showing partial inhibition effects against *E. tenella* infection upon administration of antiserum. In addition, the inhibition efficacy evaluation experiment could be repeated for several times with different batches of parasites to further verify the results in the future.

In summary, we have documented that EtMIC3 was a key molecule determining site specificity of *E. tenella*. Host cell receptors of EtMIC3 were BAG1 and ENDOUL distributed in caecal tissues of chickens. Antiserum of EtMIC3 receptor could block the infection by *E. tenella* to some extent. This result contributes greatly to elucidate the mechanism for site specificity of *Eimeria* species in natural host of chickens. In fact, several hypotheses explaining site specificity for infection by *Eimeria* species have been proposed. For example, it may be determined by the length of time during excystation and relative distribution of host cell surface molecules, even before infection takes place, suggesting intricate and multifaceted determinants of site specificity for *Eimeria* infection [7, 42, 43]. Therefore, elucidation of the exact mechanism needs further experimental evidence in future work.

## Supplementary information

**Additional file 1: Table S1. Primers for cloning of EtMICs genes.**

**Additional file 2: Table S2. Primers for cloning the potential EtMIC3 receptor genes.**

**Additional file 3: Figure S1. Analysis of the inserted fragment in chicken caecum cDNA library with PCR**. M: DL4500 marker. Lane 1-16: PCR analysis of 16 bacterial colonies.

**Additional file 4: Table S3. Identification of DNA sequences of positive prey plasmids.**

**Additional file 5: Figure S2**. **Hydrophobic profile of candidates BAG1 and ENDOUL. Prediction on hydrophobic profile of ENDOUL**. (**A**) and BAG1 (**B**) was carried out using Protean of Lasergene 8.1.3 software (DNAStar, USA). Hydrophobicity plot: the green waveform with positive value shows the hydrophobic regions, and the green waveform with negative value shows the hydrophilic regions. Alpha amphipathic regions are shown in red color and beta amphipathic regions are shown in blue color. Surface possibility: yellow waveform with positive value (> 1) shows the amino acids with high possibility on the surface.

**Additional file 6: Figure S3. Distribution of EtMIC3 receptors BAG1 and ENDOUL in chicken intestine**. Sections from upper, mid, lower intestine and caecum of chickens were incubated with antisera of EtMIC3 receptors BAG1 and ENDOUL (dilution 1:100). Subsequently, anti-rat antibody (Cy3) was used as the secondary antibody and the binding fluorescence was observed with a laser confocal microscope. **A** Weak red fluorescence was observed only in the caecum tissue incubated with BAG1 antiserum, indicating receptors BAG1 is distributed in the caecum, and not in other places of the intestine. **B** Strong red fluorescence was observed only in the caecum tissue incubated with ENDOUL antiserum, indicating receptors ENDOUL was mainly distributed in the caecum. **C** No obvious red fluorescence was observed in any intestinal section treated with pET-32a vector protein antiserum.

## Abbreviations

3-AT: 3-amino-1,2,4-triazole
ANOVA: Analysis of Variance
AMA1: Apical membrane antigen
BAG1: BCL2 associated athanogene 1
BLAST: Basic alignment search tool
BSA: Bovine Serum Albumin
DAB: 3, 3′-diaminobenzidine
*E. acervulina*: *Eimeria acervulina*
*E. coli*: *Escherichia coli*
ELISA: Enzyme-linked Immunosorbent Assay
*E. maxima*: *Eimeria maxima*
*E. mitis*: *Eimeria mitis*
ENDOUL: Endonuclease polyU-specific-like
*E. necatrix*: *Eimeria necatrix*
EtMICs: *E. tenella* MICs
GST: Glutathione S-transferase
HBSS: Hank’s Balanced Salt Solution
LB: Luria–Bertani
LGALS3: Lectin, galactoside-binding, soluble 3
IgG: Immunoglobulin G
MDBK: Bovine kidney epithelial
MICs: Microneme proteins
ORFs: Open reading frames
PBS: Phosphate Buffered Saline
PCR: Polymerase Chain Reaction
rEtAMA1: Recombinant proteins of EtAMA1
rEtMIC1: Recombinant proteins of EtMIC1
rEtMIC2: Recombinant proteins of EtMIC2
rEtMIC3: Recombinant proteins of EtMIC3
RP11-478C19.2: Anaphase promoting complex subunit 7
SD: Sprague Dawley
SDS-PAGE: Sodium dodecylsulphate polyacrylamide gel electrophoresis
SMAD5: SMAD family member 5
TBST: Tris-Buffered Saline Tween 20
UTRN: Utrophin
ZYX: Zyxin
