# Supplementary material for: EtMIC3 and its receptors BAG1 and ENDOUL are essential for site-specific invasion of Eimeria tenella in chickens
Source: Vet Res. 2020 Jul 16;51:90. doi: 10.1186/s13567-020-00809-6 (PMC7367391; doi:10.1186/s13567-020-00809-6)
Supplement: Supplementary file 1 — Additional file 1: Table S1. Primers for cloning of EtMICs genes. [file 13567_2020_809_MOESM1_ESM.docx]

**Table S1. Primers for cloning of EtMICs genes**

| Gene | Primer | Size (aa) |
| --- | --- | --- |
| EtMIC3 | Forward: 5′- CGCGGATCCATGAAGGTATACATTTGTGT-3′ | 988 |
|  | Reverse: 5′- CCGCTCGAGCTACAATGTGGCCCTC-3′ |  |
| EtMIC2 | Forward: 5′- CGGATCCATGGCTCGAGCGTTGTCGCT-3′ | 342 |
|  | Reverse: 5′- GCCTAGGTCAGGATGACTGTTGAGTGTCACTCTCT-3′ |  |
| EtMIC1 | Forward: 5′- CGGATCCATGACCAGCTCTGGCCAGG-3′ | 677 |
|  | Reverse: 5′- CAAGCTTTCATGCCCACATCTCTGATTGTT-3′ |  |
| EtAMA1 | Forward: 5′- CGCGGATCCATGCGGCGGCTTTCCCCAGC-3′ | 536 |
|  | Reverse: 5′- CCCAAGCTTCTAGTATTCCTGGTCCAGCAGCACTT-3′ |  |
