# Supplementary material for: EtMIC3 and its receptors BAG1 and ENDOUL are essential for site-specific invasion of Eimeria tenella in chickens
Source: Vet Res. 2020 Jul 16;51:90. doi: 10.1186/s13567-020-00809-6 (PMC7367391; doi:10.1186/s13567-020-00809-6)
Supplement: Supplementary file 2 — Additional file 2: Table S2. Primers for cloning the potential EtMIC3 receptor genes. [file 13567_2020_809_MOESM2_ESM.docx]

**Table S2. Primers for cloning the potential EtMIC3 receptor genes**

| Genes | Primers |
| --- | --- |
| BAG1 | F： 5' CCGGAATTCATGGCGGTTCCTGG 3'  R： 5' GCAAGCTTGTCATTCTGCCAGTGCCAAAT 3' |
| SMAD5 | F： 5' CGCGGATCCATGACGTCAATGGCCAG 3'  R： 5' TGCTCGAGTCTATGAAACAGATGAAATGG 3' |
| CTC-487M23.8 | F： 5' CGCGGATCCATGGCTGCGGCCGC 3'  R： 5' TGCTCGAGTTCATTTCTTTTTCTTCCCTT 3' |
| ENDOUL | F： 5' CCGGAATTCATGGCTGATAGGAAGGCCT 3'  R： 5' GCAAGCTTGCTAATCTTCATTACTGGTGT 3' |
| RP11-478C19.2 | F： 5' CCGGAATTCATGAGCGTGGTGGAGCACGT 3'  R：5' GCAAGCTTGTCACTGCATGCCAAACCACT 3' |
| LGALS3 | F： 5' CCGGAATTCATGTCGGACGGTTTCTCTG 3'  R：5' GCAAGCTTGTTAAATCATGGAGGTCAAAACAC 3' |
| ZYX | F：5' CGGGAATTCATGGCTTCTCCAGGTAC 3'  R：5' TGCTCGAGTTCAGCACGCTGTTTTAG 3' |
| UTRN | F：5' CCGGAATTCATGATTGGCCTGAACTT 3'  R：5' GCAAGCTTGTCACATTGCCTGTTGTCTG 3' |
