# Supplementary figures and images for: EtMIC3 and its receptors BAG1 and ENDOUL are essential for site-specific invasion of Eimeria tenella in chickens
Source: Vet Res. 2020 Jul 16;51:90. doi: 10.1186/s13567-020-00809-6 (PMC7367391; doi:10.1186/s13567-020-00809-6)

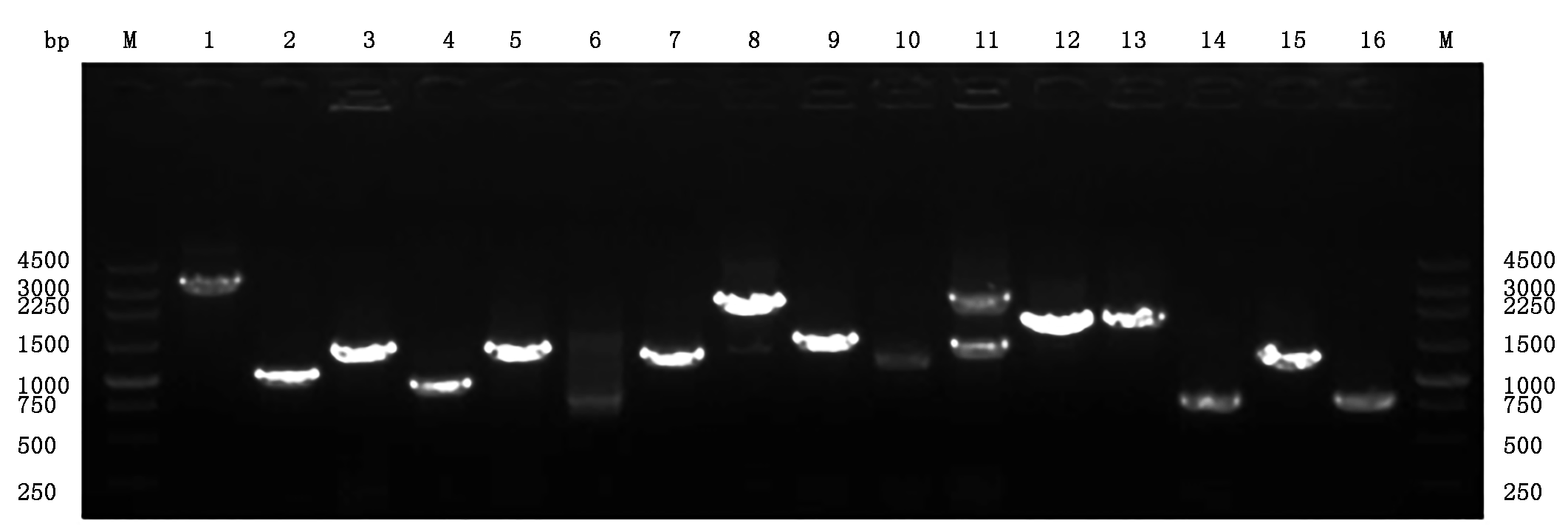

Supplement: Supplementary file 3 — Additional file 3: Figure S1. Analysis of the inserted fragment in chicken caecum cDNA library with PCR. M: DL4500 marker. Lane 1-16: PCR analysis of 16 bacterial colonies. [file 13567_2020_809_MOESM3_ESM.tif]
