# Supplementary material for: EtMIC3 and its receptors BAG1 and ENDOUL are essential for site-specific invasion of Eimeria tenella in chickens
Source: Vet Res. 2020 Jul 16;51:90. doi: 10.1186/s13567-020-00809-6 (PMC7367391; doi:10.1186/s13567-020-00809-6)
Supplement: Supplementary file 4 — Additional file 4: Table S3. Identification of DNA sequences of positive prey plasmids. [file 13567_2020_809_MOESM4_ESM.docx]

**Table S3. Identification of DNA sequences of positive prey plasmids**

| Genes | Numbers |
| --- | --- |
| RP11-478C19.2 | M4 |
| LGALS3 | M12 |
| BAG1 | M28 |
| ZYX | M74 |
| SMAD5 | M98 |
| UTRN | M103 |
| ENDOUL | M115 |
| CTC-487M23.8 | M114 |
